# Behavioural and Contextual Predictors of Cyberchondria Severity Among Preclinical Medical Students in India: A Two-Wave Longitudinal Study

**DOI:** 10.7759/cureus.110631

**Published:** 2026-06-10

**Authors:** Jaideep Rao M, Sridhar D, Kiranmai B

**Affiliations:** 1 Community Medicine, Government Medical College Maheshwaram, Ranga Reddy, IND

**Keywords:** behavioral predictors, cyberchondria, digital health literacy, india, longitudinal study, online health information seeking, medical students

## Abstract

Background

Cyberchondria, excessive online health information seeking that amplifies health anxiety, is prevalent among medical students. Longitudinal evidence on behavioural predictors of cyberchondria is sparse, particularly from India.

Objectives

The primary objective was to identify the behavioural and contextual predictors of cyberchondria severity and to assess their cross-wave stability using a repeated-measures linear mixed-effects framework among preclinical medical students. Secondary objectives were to test whether the association between online health-search frequency and cyberchondria was replicated and consistent across two waves, and to examine domain-level subscale trajectories beneath the total score.

Materials and methods

This was a prospective two-wave cohort study of 100 preclinical medical students at Government Medical College Maheshwaram, Telangana, India. The 15-item Cyberchondria Severity Scale (CSS-15) was administered at Wave 1 in January 2026 and Wave 2 in April 2026, three months apart, with no intervention between waves. The questionnaire was administered through an online survey form during scheduled department contact hours; participation was voluntary. Paired t-tests, one-way ANOVA with linear trend analysis, wave-specific multiple linear regression, and linear mixed-effects models were used, with Bonferroni and Benjamini-Hochberg false discovery rate correction for domain-level comparisons. All analyses were performed in R version 4.3.2.

Results

Online health-search frequency was the strongest independent predictor of cyberchondria across both waves (mixed-model β = 3.49, 95% CI: 2.26-4.71, p < 0.001), with a monotonic stepwise association replicated at both waves (ANOVA p < 0.001 for each). Symptom-searching before consulting a doctor (β = 3.63, 95% CI: 1.28-5.98, p = 0.002), hostel residence (β = 2.98, 95% CI: 0.38-5.57, p = 0.025), and chronic illness in self or family (β = 2.76, 95% CI: 0.56-4.96, p = 0.014) were also independently associated with higher cyberchondria severity. CSS-15 total scores were stable (paired t = -0.376, p = 0.708). Exploratory domain-level analysis suggested decreased physician mistrust (uncorrected p < 0.001; survived Bonferroni and Benjamini-Hochberg false discovery rate correction) and an increase in reassurance-seeking (uncorrected p = 0.032; did not survive Bonferroni or Benjamini-Hochberg false discovery rate correction).

Conclusions

Online health-search frequency is a robust cross-wave predictor of cyberchondria among medical students, with severity rising in a monotonic stepwise pattern across each higher category of search frequency, replicated at both time points. This finding, together with domain-level divergence in cyberchondria components, supports the evaluation of targeted digital health literacy approaches in medical education, with online health-search behaviour as a candidate focus.

## Introduction

Cyberchondria refers to excessive or repeated health-related internet searching that escalates health anxiety and distress [1,2]. It encompasses compulsive symptom-checking, difficulty disengaging from online health content, and catastrophic interpretation of ambiguous medical information [3]. The Cyberchondria Severity Scale (CSS), developed by McElroy E and Shevlin M (2014), captures this construct across five domains: Compulsion, Distress, Excessiveness, Reassurance-seeking, and Mistrust of medical professionals [1]. Online health-search behaviour, specifically the frequency, context, and purpose of health-related internet use, is consistently implicated as a proximal correlate of cyberchondria severity across studies, although the strength and consistency of this association over time remain poorly characterised [4,5].

Medical students may represent a population of particular concern because they combine increasing exposure to medical information with unrestricted access to online health content. Pre-clinical students, who possess sufficient medical vocabulary but limited clinical experience to contextualise symptom information, may be especially vulnerable to compulsive or distress-generating online health searches, although direct longitudinal evidence remains limited [6,7]. Cross-sectional studies have linked cyberchondria with health anxiety and online health information seeking, although medical-student-specific prevalence estimates vary across settings [8,9,10]. Because online health-search behaviour and cyberchondria are likely to be dynamically related, cross-sectional designs cannot confirm whether observed associations are stable over time, vary by context, or reflect bidirectional influence. This distinction matters for intervention design because predictors that show consistent associations over time may be more plausible candidates for sustained educational or behavioural targeting [11].

India presents an important context for this inquiry because of the scale of its medical education system and the broad expansion of internet connectivity and digital access, which may facilitate online health information seeking among students [12,13]. Yet longitudinal data on cyberchondria from Indian medical students are virtually absent from the published literature, and no study from India has examined whether the associations between online health-search behaviour and cyberchondria are consistent across time points, a prerequisite for confidently recommending interventions targeting search behaviour in medical curricula.

This study was therefore designed with one primary objective and two secondary objectives. The primary objective was to identify the behavioural and contextual predictors of cyberchondria severity and to assess their cross-wave stability using a unified repeated-measures linear mixed-effects framework. The two secondary objectives were (a) to test whether the association between online health-search frequency and cyberchondria, previously established only cross-sectionally, is replicated and consistent across two independent time points, and (b) to assess domain-level subscale trajectories to determine whether stability in the total score masks divergent trends in specific subscales.

A two-wave design with a three-month interval was chosen for two reasons. First, it spans one full academic block in the pre-clinical curriculum at our institution, during which first-year students consolidate Anatomy, Physiology, and Biochemistry, and second-year students progress through Pathology, Microbiology, and Pharmacology, modules that progressively expand students’ medical vocabulary and their capacity to search online for medical content. Second, it is long enough to capture potentially non-trivial within-person fluctuations in cyberchondria yet short enough to retain the same cohort within the same academic year. The scope was deliberately limited to pre-clinical first- and second-year students.

On the basis of prior cross-sectional evidence, we pre-specified four directional hypotheses: (H1) overall CSS-15 severity would remain broadly stable across the three-month interval; (H2) higher online health-search frequency would be associated with higher cyberchondria severity at both waves; (H3) searching symptoms before consulting a doctor would be independently associated with higher severity; and (H4) stability in the total score might mask divergent shifts at the domain level.

## Materials and methods

Study design, setting, and participants

This two-wave longitudinal cohort study was conducted at Government Medical College Maheshwaram (GMC Maheshwaram), a newly established government medical college located in Rangareddy district, Telangana, India, affiliated with Kaloji Narayana Rao University of Health Sciences. Data were collected at Wave 1 (January 2026) and Wave 2 (April 2026), approximately three months apart.

No educational session, behavioural intervention, or experimental manipulation was conducted between the two waves; participants continued their routine medical college curriculum during the inter-wave period. The Wave 1 and Wave 2 assessments represented repeated naturalistic measurements of the same outcome (CSS-15 score) and the same exposure variables (online health-search behaviour, internet-use duration, and symptom-searching before consultation) in the same individuals, enabling assessment of within-participant change and cross-wave consistency of predictor-outcome associations.

Ethical oversight of this study was prospective. Government Medical College Maheshwaram is a newly established institution whose Institutional Ethics Committee (IEC) was being formally constituted during the study period. The complete study protocol was submitted to the IEC on 2 January 2026, before any Wave 1 data were collected, and the committee reviewed the protocol and granted authorisation for the study to proceed before the start of Wave 1; data collection began only after this authorisation. The committee’s formal written approval (reference IEC/GMC/MAHRM/2026/01) was subsequently issued on March 24, 2026, at its first fully constituted meeting, and documented approval for the entire study period, including both waves; this was the first study approved by the newly constituted committee. Protocol submission and committee authorisation therefore both preceded Wave 1, and the March 24, 2026 date reflects the issuance of formal written documentation rather than the timing of ethical clearance. Electronic informed consent was obtained from all participants at both waves.

All first-year and second-year medical students enrolled during the study period (50 in each year; 100 in total) were invited to participate. The questionnaire was administered via a secure online survey form (Google Forms) during scheduled department contact hours; faculty facilitated distribution of the survey link and encouraged participation but did not monitor or view students’ screens or individual responses during completion. Participation remained entirely voluntary, and non-participation carried no academic consequences, as stated explicitly in the consent form and verbally at the time of administration. The survey form required a response to every item before submission, eliminating item-level missing data. Longitudinal matching across waves was performed using a combination of year of study and roll number. All 100 invited students responded with complete, matchable data at both waves; the participant-flow details are summarised in Appendix 1 (STROBE-aligned flow diagram).

Exclusion criteria

Pre-specified exclusion criteria were: (i) incomplete or duplicate responses; (ii) responses that could not be matched across waves by year of study and roll number; and (iii) a self-reported, previously diagnosed psychiatric disorder, ascertained by a single self-report screening item in the enrolment section. In practice, all 100 invited students provided complete and matchable responses at both waves, and none met an exclusion criterion; the final analytic sample therefore comprised all 100 participants. Reliance on a single self-report item to ascertain psychiatric history may under-detect relevant conditions, and this is acknowledged in the Limitations.

Privacy and matching

To protect participant privacy while enabling longitudinal linkage, each student’s year of study and institutional roll number were used only to match Wave 1 and Wave 2 records. Roll numbers were replaced with sequential anonymised participant identifiers immediately after matching, and the linkage key connecting roll numbers to anonymised identifiers was stored separately, was accessible only to the corresponding author, and was deleted after matching was verified. Student names were not used in any analysis, and the de-identified analytic dataset contained no direct identifiers.

Sample size estimation

The required sample size was estimated for a paired comparison of mean CSS-15 scores across two repeated measurements using G*Power 3.1.9.7. Assuming Cohen’s d = 0.30, two-sided α = 0.05, power = 0.80, and a within-subject correlation of 0.50, the minimum required sample was approximately 90 paired observations [14]. The final matched sample of 100 participants therefore exceeded the minimum required for the primary paired analysis; a post hoc consideration of the observed effect is provided in the Limitations.

Study instrument

We administered the CSS-15. The original Cyberchondria Severity Scale was developed by McElroy E and Shevlin M (2014) as a 33-item instrument [1] and was subsequently validated as a 15-item short form by Barke A et al. (2016) [5]. The CSS-15 was selected for its established psychometric properties, brevity facilitating repeated administration, and prior use in cross-cultural validation studies.

The CSS-15 is a publicly available, peer-reviewed research instrument, and no pre-use licensing is required for its non-commercial academic use. As a professional courtesy, we corresponded with one of the original scale developers, Dr. Eoin McElroy (Ulster University, Northern Ireland), who confirmed that he was happy for the scale to be used in this research (email correspondence, 18 May 2026). No licensing barrier applied to the conduct of the study.

Each item was administered on a 5-point frequency scale from ‘Never’ to ‘Always’ and numerically coded from 0 to 4 for analysis. The five-point verbal anchors were adapted from the frequency-based response format used in prior CSS administrations and were not independently piloted in this cohort (addressed in the Limitations). Items 5, 12, and 15 are reverse-scored. Five domain scores were computed according to the short-form CSS factor structure used in prior validation work [5]: Excessiveness (Items 1, 2, 13), Distress (Items 6, 9, 14), Compulsion (Items 3, 4, 7), Reassurance-seeking (Items 8, 10, 11), and Mistrust of medical professionals (Items 5R, 12R, 15R). Domain scores were calculated as the mean of constituent items (range 0-4). The total score is the sum of all 15 items (range 0-60), with higher scores indicating greater cyberchondria severity. Internal consistency was assessed using Cronbach’s α at each wave. The full list of CSS-15 items and response anchors is provided in Appendix 2.

Variables

The primary outcome was the CSS-15 total score at each wave; secondary outcomes were CSS-15 domain scores. Primary exposure variables were frequency of online health information searching (Rarely / Sometimes / Often / Always), average daily internet use (< 2 hours / 2-4 hours / > 4 hours), and searching for symptoms online before consulting a doctor (Yes / No). Year of study (first year vs. second year) was treated as a covariate and stratifying variable. Covariates included age, gender, place of residence (day-scholar / hostel), family setting (urban / rural), and presence of chronic illness in self or family; chronic illness, internet use, search frequency, and symptom-searching were assessed at each wave.

Statistical analysis

All statistical analyses were performed using R version 4.3.2 (R Foundation for Statistical Computing, Vienna, Austria) within the RStudio environment. Linear mixed-effects models were fitted using the lme4 package, with 95% CIs obtained using the Wald approximation. Descriptive statistics were summarised as mean ± SD for continuous variables and frequencies with percentages for categorical variables. Within-participant change scores were examined using the Shapiro-Wilk test. Paired t-tests and Wilcoxon signed-rank tests were reported for CSS-15 total and domain scores; effect sizes were reported using Cohen’s d and matched-pairs rank-biserial correlation (r_rb). Because five domain-level comparisons were conducted simultaneously, both Bonferroni correction (family-wise α = 0.05/5 = 0.01) and Benjamini-Hochberg false discovery rate (BH-FDR) correction were applied to the domain-level p-values, and domain findings are reported as exploratory.

A linear mixed-effects model with participant-level random intercepts was fitted with CSS-15 total score as the dependent variable and time, year of study, time × year, search frequency, internet-use duration, symptom-searching before consultation, gender, and age as fixed effects; an extended model additionally included residence and chronic illness. A random-slope model was compared with the random-intercept-only model by likelihood-ratio test and Akaike Information Criterion (AIC), and the simpler model was retained where the slope did not improve fit. Wald-based inferences from the mixed model were corroborated using cluster-robust sandwich standard errors clustered on the participant identifier. Four Predictor × Time interaction terms (search frequency, symptom-searching, residence, and chronic illness, each × time) were tested to evaluate whether predictor-outcome associations differed across waves. Search frequency was modelled as an ordinal predictor coded 1-4; as a sensitivity analysis, it was re-entered as an indicator-coded categorical variable (reference = Rarely). Wave-specific multiple linear regressions were fitted separately for each wave, and one-way ANOVA with linear trend analysis examined the association between search-frequency categories and CSS-15 total score at each wave. Stratified analyses by year compared within-group change using paired tests and between-group change using an independent-samples Student’s pooled-variance t-test. A sensitivity analysis used ordinary least squares (OLS) regression of change scores on baseline covariates. All regression-table values were extracted programmatically from the fitted model objects. All tests were two-sided, with α = 0.05. The de-identified dataset and analysis code are available from the corresponding author to qualified researchers upon reasonable request, subject to a data-sharing agreement and institutional ethics clearance, given the sensitive individual-level nature of the data and the re-identification risk in a small single-institution sample.

## Results

Sample characteristics

All first-year (n = 50) and second-year (n = 50) medical students were invited at both waves, and all 100 responded with complete data and were matched across waves using year and roll number. Table 1 presents Wave 1 baseline characteristics. The mean age was 19.95 ± 1.42 years; 67% were female, and 75% were from an urban family setting. At baseline, 90% used the internet for 2 hours or more daily, 72% searched for health information at least sometimes, 75% searched symptoms before consulting a doctor, and 46% reported chronic illness in self or family.

**Table 1:** Baseline socio-demographic and behavioural characteristics of participants (N = 100). MBBS: Bachelor of Medicine, Bachelor of Surgery. Family setting refers to the location of the family’s usual residence. Chronic illness denotes self-reported chronic illness in the participant or an immediate family member.

Variable	Category/Statistic	Value
Age (years)	Mean ± SD	19.95 ± 1.42
Gender	Female	67 (67.0)
	Male	33 (33.0)
Year of study	1st year MBBS	50 (50.0)
	2nd year MBBS	50 (50.0)
Residence	Day scholar	58 (58.0)
	Hostel	42 (42.0)
Family setting	Urban	75 (75.0)
	Rural	25 (25.0)
Chronic illness (self/family)	Yes	46 (46.0)
	No	54 (54.0)
Internet use (daily)	< 2 hours	10 (10.0)
	2-4 hours	49 (49.0)
	> 4 hours	41 (41.0)
Search frequency	Rarely	28 (28.0)
	Sometimes	42 (42.0)
	Often	23 (23.0)
	Always	7 (7.0)
Search before consulting a doctor	Yes	75 (75.0)
	No	25 (25.0)

Longitudinal change in CSS-15 total score

The mean CSS-15 total score was 18.07 ± 8.29 at Wave 1 (median 18.0; IQR 12.0-23.0) and 17.75 ± 8.60 at Wave 2 (median 18.0; IQR 11.0-25.0), yielding a mean change of -0.32 ± 8.51 (Table 2). This difference was not statistically significant by either parametric (paired t = -0.376, p = 0.708) or nonparametric (Wilcoxon W = 2265.0, p = 0.688) testing, and the effect size was negligible (Cohen’s d = -0.04). The Shapiro-Wilk test on within-participant change scores was significant (W = 0.959, p = 0.003), indicating a departure from normality; however, the parametric and nonparametric tests were concordant. Internal consistency was acceptable at Wave 1 (α = 0.755) and good at Wave 2 (α = 0.812). The test-retest correlation was moderate (Pearson r = 0.493, p < 0.001).

**Table 2:** Comparison of CSS-15 total and domain scores between Wave 1 and Wave 2 (N = 100). Within-participant change from Wave 1 to Wave 2 was calculated as Change = Wave 2 - Wave 1; positive values denote an increase at Wave 2. Tests included paired t-tests (parametric) and Wilcoxon signed-rank tests (nonparametric). Effect sizes are reported as Cohen’s d and matched-pairs rank-biserial correlation (r_rb). *Domain-level comparisons are secondary/exploratory; after correction for the five simultaneous domain comparisons, decreased Mistrust remained significant (Bonferroni p = 0.004; BH-FDR p = 0.004), whereas increased Reassurance-seeking did not survive correction (Bonferroni p = 0.161; BH-FDR p = 0.081). Wilcoxon signed-rank tests were concordant (Reassurance-seeking W = 1312.5, p = 0.035; Mistrust W = 548.0, p = 0.002). Domain scores are reported as the mean of constituent items (range 0-4); the total score range is 0-60. Domain means are rounded to two decimal places; the CSS-15 total is computed at full precision from raw item-level data, so reconstructing the total from the rounded domain means × 3 may differ slightly from the displayed value. BH-FDR: Benjamini-Hochberg false discovery rate.

Score	Wave 1 Mean ± SD	Wave 2 Mean ± SD	Change Mean ± SD	t	p	Cohen’s d	r_rb
CSS-15 total	18.07 ± 8.29	17.75 ± 8.60	-0.32 ± 8.51	-0.376	0.708	-0.04	-0.04
Excessiveness	1.79 ± 0.94	1.73 ± 0.92	-0.06 ± 1.00	-0.599	0.551	-0.06	-0.06
Distress	0.92 ± 0.89	1.01 ± 0.85	+0.09 ± 0.79	+1.144	0.255	+0.11	+0.12
Compulsion	0.97 ± 0.85	0.93 ± 0.80	-0.04 ± 0.96	-0.417	0.678	-0.04	-0.03
Reassurance-seeking*	1.39 ± 1.05	1.63 ± 1.09	+0.25 ± 1.14	2.172	0.032	+0.22	+0.21
Mistrust*	0.95 ± 1.06	0.61 ± 0.78	-0.34 ± 0.99	-3.465	<0.001	-0.35	-0.32

Domain-level changes

Although the CSS-15 total score remained stable, domain-level analysis, interpreted as exploratory and corrected for multiple comparisons, showed that components did not all change in the same direction. Mistrust of medical professionals decreased from 0.95 ± 1.06 to 0.61 ± 0.78 (uncorrected p < 0.001), a change that remained significant after both Bonferroni and BH-FDR correction. Reassurance-seeking increased modestly from 1.39 ± 1.05 to 1.63 ± 1.09 (uncorrected p = 0.032), but this did not survive correction for multiple comparisons and is therefore reported as exploratory. Excessiveness, Distress, and Compulsion did not change significantly. These findings suggest that total scores alone may not fully capture shifts in the domain profile of cyberchondria over time, although the domain-level results require cautious, exploratory interpretation.

Stratified analysis by year of study

**Figure 1:**
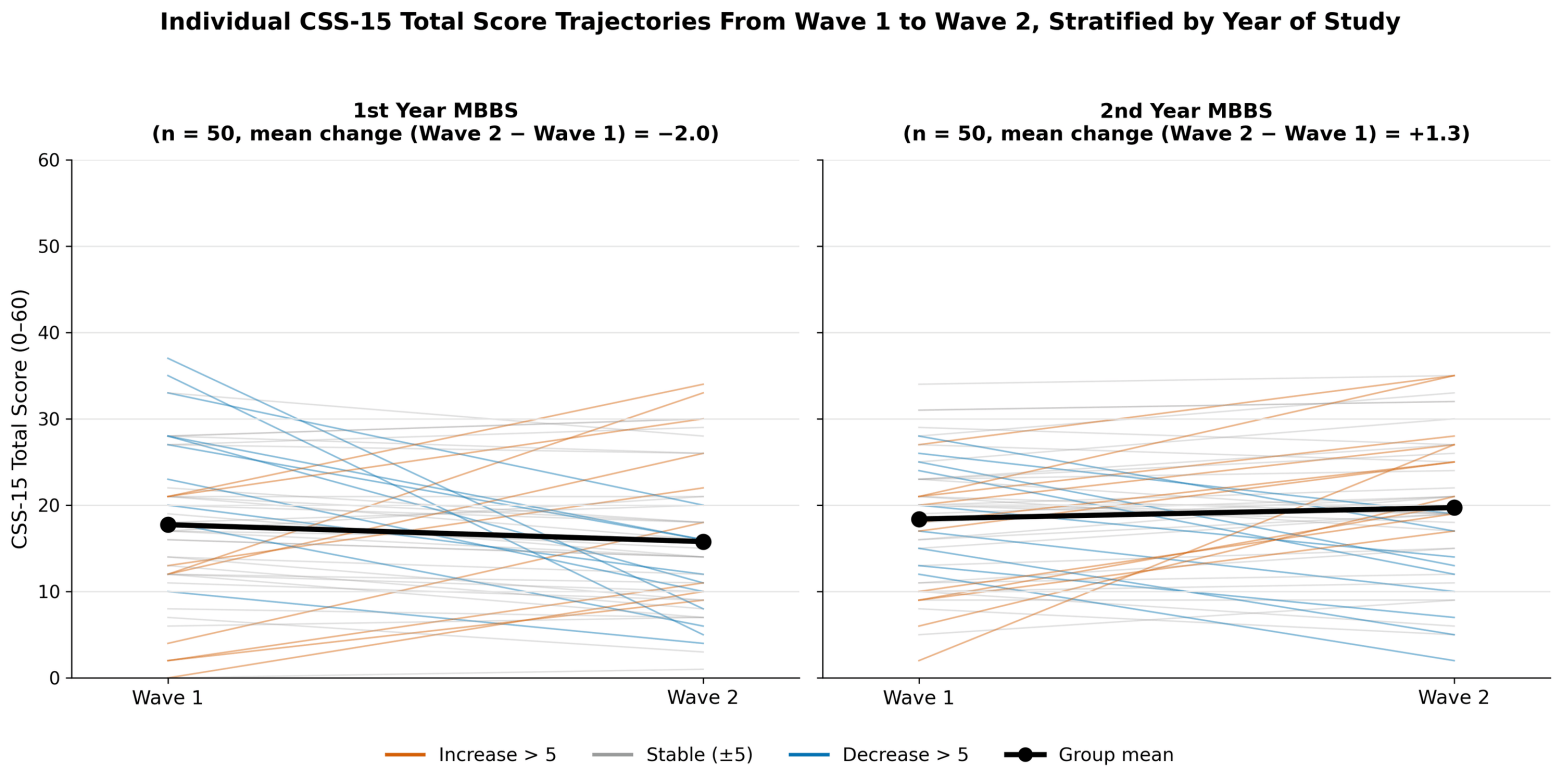
Individual CSS-15 total score trajectories from Wave 1 to Wave 2, stratified by year of study. Each thin line represents one participant; the thick black line represents the group mean. Lines are colour-coded by the direction and magnitude of within-person change (Wave 2 - Wave 1): orange = increase >5 points; blue = decrease >5 points; grey = stable (±5 points). The ±5-point threshold is a descriptive cut-off for visualisation only and has no clinical or psychometric basis. The y-axis spans the full CSS-15 range (0-60). Within-group changes were not statistically significant (first year: p = 0.148; second year: p = 0.201), and the between-group difference in change scores was not statistically significant (t = -1.955, p = 0.053). Substantial between-participant heterogeneity is visible beneath the stable group-level means (random-intercept variance = 21.26; ICC: 0.423). CSS-15: Cyberchondria Severity Scale-15; ICC: Intraclass correlation coefficient.

First-year students showed a non-significant change in CSS-15 total score, from 17.74 ± 9.08 to 15.78 ± 8.40 (change -1.96 ± 9.43; p = 0.148), as did second-year students, from 18.40 ± 7.48 to 19.72 ± 8.43 (change +1.32 ± 7.20; p = 0.201). The between-group difference in change scores was not statistically significant (t = -1.955, p = 0.053); the null hypothesis of no cohort-level differential change cannot be rejected (Figure 1).

Monotonic stepwise association between search frequency and cyberchondria

A monotonic stepwise association was observed between health-search frequency and CSS-15 total score at both waves. At Wave 1, mean CSS-15 scores increased progressively from 11.5 (Rarely, n = 28) to 19.0 (Sometimes, n = 42), 22.2 (Often, n = 23), and 25.1 (Always, n = 7); one-way ANOVA F = 13.06, p < 0.001; linear trend r = 0.516, p < 0.001. At Wave 2, a similar gradient was observed: 11.4 (Rarely, n = 21), 18.3 (Sometimes, n = 47), 20.0 (Often, n = 23), and 23.9 (Always, n = 9); ANOVA F = 7.05, p < 0.001; linear trend r = 0.397, p < 0.001. The consistency of this stepwise gradient across both time points supports a robust association.

Within-person behavioural migration

Although search-frequency category proportions were broadly similar across waves at the group level, substantial movement occurred within individuals. Of the 100 participants, 53 (53%) changed search-frequency category between waves: 32 moved to a higher-frequency category and 21 to a lower-frequency category, while 47 (47%) remained in the same category. The full Wave 1 → Wave 2 transition matrix is provided in Appendix 3. This individual-level mobility, masked by stable group proportions, reinforces the value of the repeated-measures design.

Bivariate associations of behavioural and contextual variables

Searching symptoms before consulting a doctor was associated with higher CSS-15 scores at both waves: Wave 1 (Yes, n = 75: 19.44 ± 7.70 vs. No, n = 25: 13.96 ± 8.76; t = 2.975, p = 0.004) and Wave 2 (Yes, n = 69: 19.91 ± 7.89 vs. No, n = 31: 12.94 ± 8.27; t = 4.029, p < 0.001). Hostel residence was associated with higher scores at Wave 1 (Hostel: 20.19 ± 8.05 vs. Day-scholar: 16.53 ± 8.18; t = 2.221, p = 0.029) but not at Wave 2 (p = 0.142). Chronic illness in self or family was associated with higher scores at both waves at the bivariate level: Wave 1 (Yes: 20.22 ± 8.38 vs. No: 16.24 ± 7.83; t = 2.452, p = 0.016) and Wave 2 (Yes: 20.38 ± 8.81 vs. No: 15.12 ± 7.61; t = 3.196, p = 0.002). Independent-samples comparisons used Student’s pooled-variance t-tests.

Linear mixed-effects model

Table 3 presents results from the extended linear mixed-effects model with participant-level random intercepts, including residence and chronic illness as additional covariates. A random-slope model was tested but did not improve fit (likelihood-ratio χ² = 0.63, df = 2, p = 0.731; AIC difference = 3.4 favouring the intercept-only model); the random-intercept model was therefore retained. Four variables were independently associated with higher cyberchondria severity across both waves. Search frequency was the strongest predictor (β = 3.49, 95% CI: 2.26 to 4.71, p < 0.001): each unit increase in search-frequency category was associated with a 3.49-point higher CSS-15 total score. Symptom-searching before consulting a doctor was independently associated with a 3.63-point increase (β = 3.63, 95% CI: 1.28 to 5.98, p = 0.002). Hostel residence was associated with a 2.98-point higher score (β = 2.98, 95% CI: 0.38 to 5.57, p = 0.025), and chronic illness in self or family was associated with a 2.76-point increase (β = 2.76, 95% CI: 0.56 to 4.96, p = 0.014). The main effect of time was not significant (β = -1.60, p = 0.146), confirming overall temporal stability, and the time × year interaction was not significant (β = 1.94, p = 0.207). Gender, age, and internet-use duration were not significant. None of the four Predictor × Time interaction terms was statistically significant (all p > 0.10; joint Wald test p = 0.473), indicating that predictor-outcome associations did not differ significantly across waves; Wald-based inferences were corroborated by cluster-robust standard errors. The random-intercept variance was 21.26 (ICC = 0.423). Full fixed-effect estimates, including the non-significant covariates and the intercept, are reported in Appendix 4.

**Table 3:** Extended linear mixed-effects model for CSS-15 total score (200 observations from 100 participants). †Search frequency was coded as follows: Rarely = 1, Sometimes = 2, Often = 3, and Always = 4. Chronic illness in self or family was assessed at each wave and modelled as a time-varying covariate. None of the Predictor × Time interaction terms was statistically significant (joint Wald test p = 0.473). The ICC was 0.423 (random-intercept variance = 21.26; residual variance = 29.00). Gender, age, and internet-use duration were non-significant; full estimates for all terms are provided in Appendix 4. Wald-based inferences were corroborated by cluster-robust standard errors. SE: Standard error; ICC: Intraclass correlation coefficient; CSS-15: Cyberchondria Severity Scale-15.

Fixed effect	β	SE	95% CI	z	p-value
Time (Wave 2 vs. Wave 1)	-1.6	1.1	-3.75 to 0.56	-1.45	0.146
Year (2nd vs. 1st)	-2.11	1.54	-5.13 to 0.91	-1.37	0.171
Time × Year	1.94	1.54	-1.08 to 4.96	1.26	0.207
Search frequency†	3.49	0.63	2.26 to 4.71	5.58	<0.001
Search before doctor	3.63	1.2	1.28 to 5.98	3.03	0.002
Residence (hostel vs. day scholar)	2.98	1.32	0.38 to 5.57	2.25	0.025
Chronic illness (yes vs. no)	2.76	1.12	0.56 to 4.96	2.45	0.014

Wave-specific regression models

Table 4 presents wave-specific multiple linear regression models, provided as a descriptive cross-sectional summary of associations at each wave. Because the corresponding Predictor × Time interactions in the mixed model were not statistically significant, differences between the Wave 1 and Wave 2 coefficients should not be interpreted as evidence of true temporal change. Search frequency was statistically significant at both waves. Symptom-searching reached adjusted statistical significance at Wave 2 but not at Wave 1, although it was significant at both waves in the bivariate analysis. Hostel residence was significant at Wave 1 and chronic illness at Wave 2 in the adjusted wave-specific models; given the non-significant interaction terms, these wave-specific differences are descriptive only. Full model estimates are provided in Appendix 5.

**Table 4:** Wave-specific multiple linear regression models for CSS-15 total score. Model fit: Wave 1 R² = 0.403 (adjusted R² = 0.336, overall F-test p < 0.001); Wave 2 R² = 0.331 (adjusted R² = 0.256, overall F-test p < 0.001). The 95% CIs use the Wald approximation (β ± 1.96 × SE); t = β/SE. Symptom-searching was significant at both waves in the bivariate analysis (Wave 1 p = 0.004; Wave 2 p < 0.001) but reached adjusted significance only at Wave 2. This table is a descriptive cross-sectional summary; because the Predictor × Time interactions in the mixed model were not statistically significant, wave-specific coefficient differences should not be interpreted as temporal change. SE: Standard error; CSS-15: 15-item Cyberchondria Severity Scale.

Predictor	Wave 1 β (95% CI)	Wave 1 t	Wave 1 p	Wave 2 β (95% CI)	Wave 2 t	Wave 2 p
Search frequency	4.55 (2.88 to 6.22)	5.35	<0.001	2.38 (0.42 to 4.34)	2.38	0.02
Search before consulting a doctor	2.55 (-0.80 to 5.90)	1.49	0.14	4.64 (1.19 to 8.09)	2.64	0.01
Year of study (2nd vs. 1st)	-2.49 (-5.55 to 0.57)	-1.6	0.113	0.17 (-3.30 to 3.64)	0.1	0.922
Gender (female vs. male)	0.86 (-2.28 to 4.00)	0.54	0.593	0.10 (-3.33 to 3.53)	0.06	0.956
Residence (hostel vs. day scholar)	4.39 (0.92 to 7.86)	2.48	0.015	1.71 (-2.01 to 5.43)	0.9	0.37
Chronic illness (yes vs. no)	1.65 (-1.21 to 4.51)	1.13	0.26	3.64 (0.62 to 6.66)	2.36	0.021

Sensitivity and supplementary analyses

In the sensitivity analysis using OLS regression of the change score on baseline covariates (R² = 0.146, F = 2.653, p = 0.020), higher baseline search frequency predicted a greater decline in CSS-15 score over time (β = -2.75, p = 0.006). This pattern is consistent with regression toward the mean among high-baseline searchers and is a distinct, non-competing phenomenon from the stable cross-wave search-frequency-severity association identified in the mixed model. The year-of-study coefficient was not statistically significant (β = 3.58, p = 0.053). No statistically significant within-participant changes were observed in search frequency (Wilcoxon p = 0.250), internet-use duration (Wilcoxon p = 0.083), or symptom-searching (McNemar p = 0.286) between waves, although internet-use duration was numerically higher at Wave 2 (> 4 hours daily: 41% at Wave 1 vs. 54% at Wave 2). Complete regression diagnostics for the extended mixed-effects model are provided in Appendix 6, the corresponding residual diagnostic plots in Appendix 7, and a sensitivity analysis with search frequency entered as an indicator-coded categorical variable in Appendix 8.

## Discussion

Principal findings

This study adds longitudinal evidence on cyberchondria among Indian medical students, showing that overall CSS-15 total scores were stable over three months while selected domains changed in different directions: mistrust of physicians decreased, remaining significant after correction, and reassurance-seeking increased, although this was exploratory and did not survive correction, whereas other domains were stable. The most consistent cross-wave associations were with search frequency (β = 3.49, p < 0.001) and symptom-searching before consultation (β = 3.63, p = 0.002). Hostel residence (β = 2.98, p = 0.025) and chronic illness in self or family (β = 2.76, p = 0.014) were also independently associated in the mixed model; wave-specific analyses showed residence reaching significance at Wave 1 and chronic illness at Wave 2, but because the Predictor × Time interactions were not significant, these wave-specific differences are descriptive rather than evidence of temporal change. Differences in change between first- and second-year students were not statistically significant.

Practical implications of domain-level assessment

The contrast between stable total scores and changing domain profiles highlights that cyberchondria may be better understood as a multidimensional construct. Total-score-only interpretation would suggest little change, whereas domain-level findings indicate modest shifts in mistrust and reassurance-seeking. From an intervention perspective, assessing specific domains alongside total severity may be more informative, although the domain-level results should be read as exploratory.

Comparison with existing literature

The observed temporal stability is consistent with trait-like conceptualisations of cyberchondria [15]. The moderate test-retest correlation (r = 0.493) is broadly consistent with the partial temporal stability reported in CSS validation work over comparable intervals [1]. The stepwise association between search frequency and cyberchondria aligns with cross-sectional findings from Turkey [16], Egypt [17], and Germany [5]; the present study extends these findings by showing that the gradient was reproduced at both waves and remained significant in the mixed model after adjustment. The association with pre-consultation symptom-searching is consistent with theoretical accounts linking repeated symptom-checking to anxiety, although our observational design cannot establish the proposed mechanisms of confirmation bias or catastrophic interpretation [3,18].

The association of hostel residence with cyberchondria at Wave 1 and of chronic illness at Wave 2 in the wave-specific models is descriptive; because the corresponding interaction terms were not statistically significant, we do not interpret these as genuine temporal shifts, and any developmental explanation would be speculative. These patterns nonetheless illustrate the value of examining contextual correlates within a longitudinal design.

Public health and curricular implications

The consistent cross-wave association between search frequency and cyberchondria identifies search behaviour as a potentially modifiable correlate: in the mixed model, each higher search-frequency category was associated with a 3.49-point higher CSS-15 score. Comparisons of the extreme ‘Always’ versus ‘Rarely’ groups are based on small subgroups, for example, only seven ‘Always’ searchers at Wave 1, and are therefore less stable than the overall mixed-model estimate. Because the most robust finding was the search-frequency association, and the only corrected domain-level change was decreased Mistrust, rather than changes in the Excessiveness or Distress domains, curricular efforts may be best directed at structured appraisal of online health information and at reinforcing the observed improvement in physician trust. Medical colleges may consider integrating structured digital health literacy modules into the pre-clinical curriculum; eHealth-literacy frameworks offer one candidate basis for such modules, although their effectiveness in this population has not been established [19,20]. Domain-level assessment may add value to any future cyberchondria screening within student wellbeing programmes.

Strengths and limitations

Strengths include the prospective longitudinal repeated-measures design with complete matched follow-up of all 100 participants; concordant parametric and nonparametric analyses; reporting of both Cohen’s d and rank-biserial effect sizes; formal Predictor × Time interaction testing; cluster-robust corroboration of the Wald-based inferences; and a focus on pre-clinical medical students, an at-risk group underrepresented in the longitudinal literature. This is one of the first longitudinal studies of cyberchondria among Indian medical students.

This study has several limitations. First, it was conducted at a single, newly established institution, which limits generalisability. Second, the matched sample of 100 paired observations provided adequate power for the primary paired comparison but limited power for subgroup and interaction analyses; the observed total-score effect was negligible (d = −0.04), and the non-significant change should be read as a failure to reject the null rather than as positive evidence of stability. Third, the two-wave, three-month design captures only a short trajectory and precludes growth-curve modelling. Fourth, exposure and outcome were measured contemporaneously by self-report, so associations may reflect social desirability or recall bias and cannot establish causation. Fifth, although faculty did not view individual responses and participation was voluntary, administration during faculty-facilitated contact hours means that perceived non-voluntariness and social desirability bias cannot be excluded. Sixth, psychiatric history was ascertained by a single self-report item, which may under-detect relevant conditions. Seventh, the verbal response anchors were adapted from prior CSS administrations but were not independently piloted in this cohort. Eighth, the CSS-15 factor structure was adopted from prior validation work and was not revalidated by confirmatory factor analysis in this Indian sample; item 15, in particular, may capture heterogeneous content, warranting cautious interpretation of the Mistrust domain. Ninth, five domain-level comparisons were conducted; although Bonferroni and BH-FDR corrections were applied, the domain-level findings remain exploratory. Tenth, none of the Predictor × Time interactions was significant, so wave-specific coefficient differences are descriptive only. Eleventh, core psychopathology constructs that frequently co-occur with cyberchondria, such as health anxiety, intolerance of uncertainty, and obsessive-compulsive features, were not measured, limiting construct triangulation. Twelfth, the two waves coincided with different points in the academic calendar, a potential confounder of within-person change. Findings should therefore be interpreted as associations rather than causal effects. Future multi-institutional studies with three or more waves, larger samples, longer follow-up, and concurrent psychopathology measures are recommended.

## Conclusions

Among pre-clinical medical students in India, overall cyberchondria severity remained stable over three months, while domain-level analyses suggested modest, exploratory shifts, most notably a significant decrease in mistrust of physicians and a non-robust increase in reassurance-seeking. Online health-search frequency showed a monotonic stepwise association with cyberchondria severity that was replicated at both waves and was the strongest independent predictor in the mixed-effects model. Symptom-searching before medical consultation, hostel residence, and chronic illness in self or family were also independently associated with higher cyberchondria scores. Because predictor-outcome associations did not differ significantly across waves, wave-specific differences are descriptive; the overall pattern nonetheless underscores the value of repeated-measures designs and domain-level assessment in cyberchondria research. These findings identify online health-search behaviour and specific behavioural dimensions as candidate foci for digital health literacy approaches that warrant evaluation in future controlled studies, rather than supporting reliance on total scores alone.

## Figures and Tables

**Figure 2:**
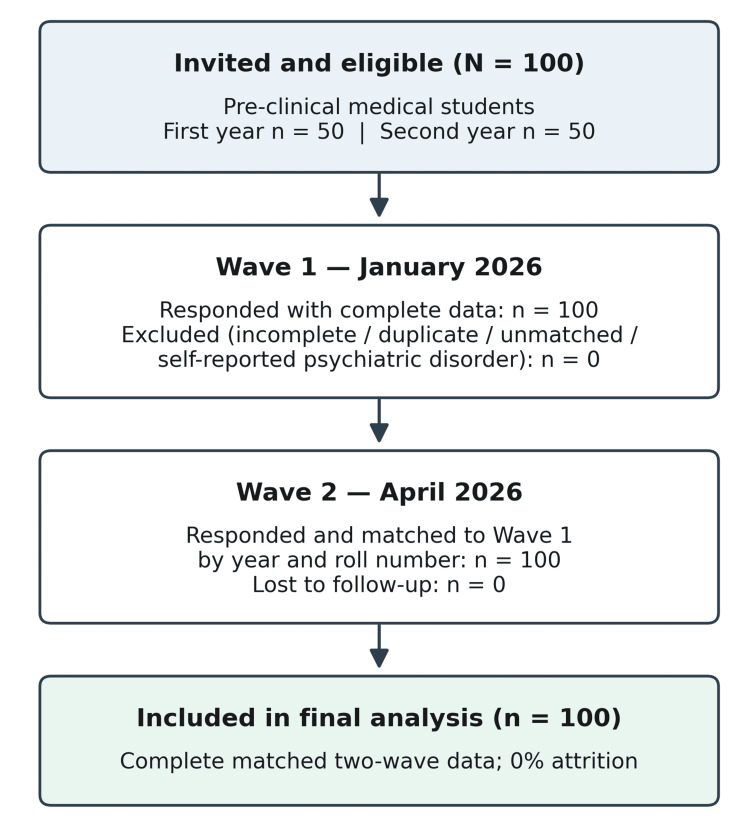
STROBE-aligned participant flow diagram. STROBE: Strengthening the Reporting of Observational Studies in Epidemiology.

**Figure 3:**
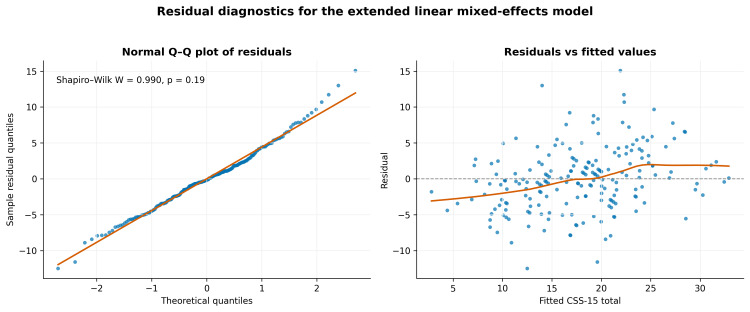
Residual diagnostics for the extended mixed-effects model: Normal Q-Q plot and residuals-versus-fitted plot.

## Appendices

Invited and eligible

100 pre-clinical medical students (first year, n = 50; second year, n = 50).

→ Responded at Wave 1 (January 2026) with complete data: 100. → Excluded because of incomplete, duplicate, unmatched responses or self-reported psychiatric disorder: 0. → Responded at Wave 2 (April 2026) and matched to Wave 1 by year and roll number: 100. → Included in final analysis: 100 (0% attrition).

Appendix 1

Appendix 2

CSS-15 Items and Response Anchors

All items were administered on a 5-point frequency scale: Never (0), Rarely (1), Sometimes (2), Often (3), and Always (4). Items 5, 12, and 15 are reverse-scored. The total score is the sum of all 15 items, with a possible range of 0-60.

1. If I notice an unexplained bodily symptom, I will search for it on the internet (e.g., a cough, headache, rash, lump, or pain somewhere). 2. I look for the same symptoms on the internet. 3. The internet search for information about symptoms or a suspected disease disturbs my search for other online information (e.g., my work, studies, or school). 4. The internet search for information about symptoms or a suspected disease disrupts my online leisure activities (e.g., streaming movies). 5. I give more importance to my doctor's assessment than my online research. (reverse-scored) 6. I panic when I read online that a symptom I have is a rare or serious condition. 7. The internet search for information about symptoms or a suspected disease disrupts my work on the computer (e.g., writing emails, working on documents, or doing calculations). 8. I discuss the results of my online research with my family doctor or pharmacist. 9. After looking for information about symptoms or a suspected disease, I feel more anxious and stressed than before. 10. The internet search for information about symptoms or a suspected disease leads me to a specialist. 11. It soothes me to discuss the online information about a suspected disease with my family doctor. 12. I trust the diagnosis of my family doctor more than my own online self-diagnosis. (reverse-scored) 13. When I search for symptoms or a disease online, I visit both trustworthy sites and/or lay forums. 14. After looking for information about symptoms or a suspected disease, I have difficulty falling asleep. 15. If my family doctor considers the results of my own online research to be wrong, I stop worrying about it. (reverse-scored)

Appendix 3

**Table 5:** Wave 1 → Wave 2 search-frequency transition matrix (N = 100). Diagonal cells represent participants who remained in the same category (47 stable). Off-diagonal cells represent participants who changed category (53 total: 32 moved to a higher category and 21 moved to a lower category).

Wave 1 ↓ / Wave 2 →	Rarely	Sometimes	Often	Always	Wave 1 total
Rarely	14	11	3	0	28
Sometimes	5	25	10	2	42
Often	2	8	7	6	23
Always	0	3	3	1	7
Wave 2 total	21	47	23	9	100

Appendix 4 

**Table 6:** Complete fixed-effect estimates from the extended mixed-effects model. Random-intercept variance = 21.26; residual variance = 29.00; ICC = 0.423. Search frequency was coded from 1 to 4 (Rarely to Always); internet-use duration was coded from 1 to 3 (<2 h, 2-4 h, >4 h); and chronic illness was assessed at each wave as a time-varying covariate. The 95% CI represents the Wald CI. ICC: Intraclass correlation coefficient

Fixed effect	β	SE	95% CI	z	p
Intercept	-6	10.51	-26.60 to 14.61	-0.57	0.568
Time (Wave 2 vs. Wave 1)	-1.6	1.1	-3.75 to 0.56	-1.45	0.146
Year of study (2nd vs. 1st)	-2.11	1.54	-5.13 to 0.91	-1.37	0.171
Time × Year of study	1.94	1.54	-1.08 to 4.96	1.26	0.207
Search frequency (1-4)	3.49	0.63	2.26 to 4.71	5.58	<0.001
Internet-use duration (1-3)	0.23	0.76	-1.25 to 1.71	0.3	0.764
Search before consulting a doctor (yes vs. no)	3.63	1.2	1.28 to 5.98	3.03	0.002
Gender (female vs. male)	0.37	1.4	-2.38 to 3.11	0.26	0.795
Age (years)	0.59	0.52	-0.42 to 1.61	1.15	0.252
Residence (hostel vs. day scholar)	2.98	1.32	0.38 to 5.57	2.25	0.025
Chronic illness (yes vs. no)	2.76	1.12	0.56 to 4.96	2.45	0.014

Appendix 5 

**Table 7:** Complete fixed-effect estimates - Panel A: extended mixed-effects model; Panel B: wave-specific multiple linear regression models (Wave 1 and Wave 2). Complete fixed-effect estimates from the extended mixed-effects model and the wave-specific multiple linear regression models are shown. The outcome was the CSS-15 total score; N = 100 per wave. All entered covariates are included, including both significant and non-significant predictors. β = unstandardised coefficient; SE = standard error; 95% CI = Wald confidence interval (β ± 1.96 × SE); t = β/SE. Reference categories: 1st-year MBBS, male, day-scholar, no chronic illness, urban setting, Hindu, <2 hours of internet use, and no symptom-searching before consulting a doctor. Search frequency was coded 1-4 (rarely-always), and internet-use duration was coded 1-3. Wave 1: R² = 0.403, adjusted R² = 0.336, F(10, 89) = 6.02, p < 0.001. Wave 2: R² = 0.331, adjusted R² = 0.256, F(10, 89) = 4.41, p < 0.001. Wave 1 and Wave 2 coefficient differences are descriptive only; no predictor × time interaction in the mixed-effects model (Table 3) was significant.

Predictor	Wave 1 β (SE)	Wave 1 95% CI	W1 t	W1 p	Wave 2 β (SE)	Wave 2 95% CI	W2 t	W2 p
Intercept	-0.87 (12.32)	-25.35 to 23.61	-0.07	0.944	-14.36 (13.36)	-40.91 to 12.19	-1.07	0.285
Search frequency	4.55 (0.85)	2.87 to 6.24	5.38	<0.001	2.38 (1.00)	0.39 to 4.36	2.37	0.02
Symptom-search before doctor	2.55 (1.71)	-0.85 to 5.96	1.49	0.14	4.64 (1.76)	1.14 to 8.15	2.63	0.01
Year (2nd vs 1st)	-2.49 (1.56)	-5.58 to 0.60	-1.6	0.113	0.17 (1.77)	-3.35 to 3.70	0.1	0.922
Gender (female vs male)	0.86 (1.60)	-2.32 to 4.03	0.54	0.593	0.10 (1.75)	-3.39 to 3.58	0.05	0.956
Residence (hostel vs day-scholar)	4.39 (1.77)	0.88 to 7.91	2.48	0.015	1.71 (1.90)	-2.07 to 5.50	0.9	0.37
Chronic illness (yes vs no)	1.65 (1.46)	-1.24 to 4.54	1.13	0.26	3.64 (1.54)	0.57 to 6.70	2.36	0.021
Age (years)	0.23 (0.60)	-0.96 to 1.42	0.38	0.707	0.99 (0.65)	-0.31 to 2.29	1.51	0.134
Family setting (rural vs urban)	0.74 (1.86)	-2.96 to 4.43	0.4	0.693	1.23 (2.06)	-2.87 to 5.32	0.6	0.553
Internet-use duration (1-3)	0.09 (1.09)	-2.08 to 2.26	0.08	0.934	0.35 (1.16)	-1.95 to 2.66	0.3	0.763
Religion (Muslim vs Hindu)	3.02 (1.79)	-0.54 to 6.57	1.68	0.096	0.41 (1.94)	-3.45 to 4.28	0.21	0.832

Appendix 6

**Table 8:** Regression diagnostics for the extended mixed-effects model. VIF: Variance inflation factor.

Diagnostic	Result	Reference threshold	Interpretation
Multicollinearity (VIF)	Range: 1.05-3.04; substantive predictors ≤1.52	<5	No problematic collinearity
Residual normality (Shapiro-Wilk)	W = 0.990, p = 0.19	p > 0.05	Residuals were approximately normal
Influential observations (Cook’s distance)	Maximum = 0.097; 4 cases >4/N (0.040)	<1.0; 4/N as a screening threshold	No highly influential observations by the conventional <1.0 threshold; however, 4 cases exceeded the 4/N screening threshold and were flagged for review

Multicollinearity

Variance inflation factors (VIFs) ranged from 1.05 to 3.04; VIFs for the substantive predictors (search frequency, internet-use duration, symptom-searching, gender, age, residence, and chronic illness) were all ≤1.52, with the higher values (2.0-3.0) confined to the time, year, and time × year terms, reflecting the expected structural collinearity introduced by the interaction. All VIFs were below the conventional threshold of 5.

Residual normality 

The Shapiro-Wilk test on the model residuals was non-significant (W = 0.990, p = 0.19), and the normal Q-Q plot showed no material departure from normality.

Influential observations 

Leave-one-participant-out Cook’s distances were computed for all 100 participants. The maximum Cook’s distance was 0.097, well below the conventional cutoff of 1.0; although four participants marginally exceeded the stringent 4/N reference value (0.040), none approached a level indicating undue influence on the model estimates.

Appendix 7

Appendix 8 

**Table 9:** Sensitivity analysis: Indicator-coded search frequency (reference category = rarely). Estimates are from the extended mixed-effects model with search frequency entered as an indicator-coded categorical predictor, adjusted for all covariates listed in Table 3. The monotonic increase across categories supports the ordinal linear coding used in the main analysis.

Search-frequency category	β	SE	95% CI	p
Sometimes vs rarely	6.48	1.28	3.96 to 9.00	<0.001
Often vs rarely	8.84	1.51	5.87 to 11.81	<0.001
Always vs rarely	9.94	2.06	5.90 to 13.99	<0.001
